# Which are the options and dosages for clobazam shortages on epilepsy treatment? Review of literature and survey of specialists

**DOI:** 10.1055/s-0045-1811235

**Published:** 2025-09-08

**Authors:** Lécio Figueira Pinto, Guilherme Simone Mendonça, Carlos Alberto Mantovani Guerreiro

**Affiliations:** 1 Universidade de São Paulo, Faculdade de Medicina, São Paulo SP, Brazil.; 2 Hospital Santa Isabel, Blumenau SC, Brazil.; 3 Universidade Estadual de Campinas, Faculdade de Ciências Médicas, Campinas SP, Brazil.

**Keywords:** Clobazam, Epilepsy, Dosage, Surveys and Questionnaire, Review

## Abstract

Clobazam (CLB) has been an established treatment for epilepsy since the 1970s, with a broad spectrum. It is frequently used as add on therapy for refractory patients. Furthermore, it is different from classic benzodiazepines (BZD) for containing nitrogen atoms in 1 and 5 positions of B ring (other are 1.4-BZD). This explains why CLB has a better tolerability and a lower chance of causing sedation, being an excellent option for epilepsy treatment compared with other BZDs. Evidence argues against the development of CLB tolerance in most patients. Antiseizure medication shortages have been reported by many countries, including the one studied here. Shortages make treatment harder, increase the need for extra clinical appointments, for orientation and medication changes, increase medication errors, decrease adherence, and cause insecurity. A literature review showed scarce evidence of alternatives, with a wide variation in dosage equivalence. A survey of specialists revealed that switch appropriateness was deemed inadequate by the majority, due to risk of seizure worsening and side effects. Clonazepam and nitrazepam were the most used BZDs, but there was great variation for clonazepam dosages (0.25–2 mg, commonly 1 per 10 mg of CLB). Better consensus was obtained for nitrazepam (5 per 10 mg of CLB). Gradual tapering of CLB, with concomitant increase of clonazepam or nitrazepam under close supervision, is advised. It is important to assess tolerability and the need for increased dosage. As CLB is an essential tool in the epilepsy armamentarium, shortages pose great risk to the patients. Governments and society must create mechanisms to prevent shortages of critical and unique medications.

## INTRODUCTION

Benzodiazepines (BZD) are well established treatments for epilepsy. There are many some options, with different formulations, indications and availability worldwide. The most frequent ones are clobazam (CLB), clonazepam, nitrazepam, diazepam and lorazepam. 1

Although CLB (7-chloro-1-methyl-5-phenyl-1.5-benzodiazepine) was originally established as a nonsedative agent to treat anxiety in the 1970s, 2 it has also been used for treatment of epilepsy, since 1974. Furthermore, in 1978, Gastaut described its efficacy as an antiseizure medication “in a percentage that has so far never been attained with any other antiepileptic agent.” 3 4

It has been widely used on the treatment of epilepsy for over 50 years and is available in over 100 countries. 5

Furthermore, CLB has broad-spectrum antiseizure effect, for both focal and generalized and seizures, and became a very popular treatment option over the world. There is trend for its choice over clonazepam (both are the most used oral BZD for epilepsy treatment). 6

## DIFFERENCES FROM OTHER BENZODIAZEPINES

**Figure 1:**
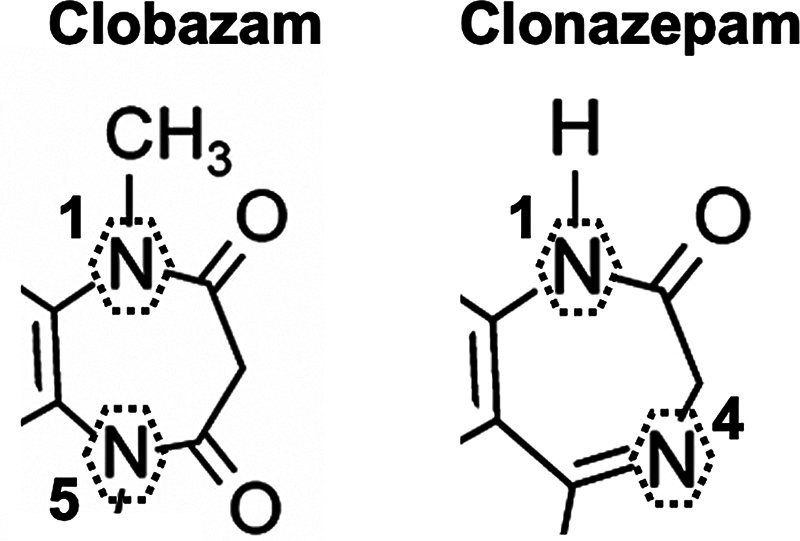
Structural differences of clobazam to other 1,4-benzodiazepines, here clonazepam as an example. Adapted from: Gauthier and Mattson. 2

There is a difference between CLB and the classic BZD because it contains nitrogen atoms in the 1 and 5 positions of the B ring (other are 1.4-BZDs), as shown in Figure 1 .

Furthermore, CLB and other BZD can bind allosterically to the GABAA receptor and increase the frequency of chloride channel opening, allowing chloride to enter and hyperpolarize the neuron. 2

**Figure 2:**
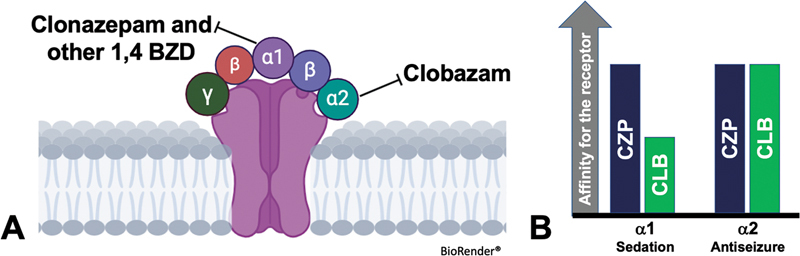
Differences of clobazam to clonazepam and other 1.4-benzodiazepines in ( **A** ) the GABAA receptor subunit selectivity and ( **B** ) the consequences of these differences in efficacy and tolerability. Adapted from: Gauthier and Mattson, Sankar et al., and Perucca et al. 2 7 8

Due to this difference, CLB has a different binding property. The GABAA receptors are composed of five subunits (usually two α, two β, and one γ). While CLB has a greater selectivity for α2, 1.4-BZDs are more selective for α1 ( Figure 2A ). 2 7 8

This difference explains why CLB has improved tolerability, with less sedation and better profile for epilepsy treatment compared with the other 1.4-BZDs (clonazepam, nitrazepam, diazepam etc.), as shown in Figure 2B . 2 9 10 11 12

## OTHER FACTS ABOUT CLOBAZAM

The metabolism of this drug occurs by liver via cytochrome P450 pathway. The enzyme CYP2C19 demethylates CLB to the pharmacodynamically active N-desmethylclobazam, which has long half-life (∼50 hours), over twice CLB's. Enzyme inducers, such as carbamazepine, phenobarbital, and phenytoin, increase CLB's clearance due to induction of CYP3A4, lowering its concentration in more than 50%. Antiseizure medications such as cannabidiol, stiripentol, and cenobamate are CYP2C19 inhibitors, thus increasing levels N-desmethylclobazam. As it is therapeutically active and has a long half-life, this can be responsible for both increased efficacy and side effects. 2 13 14 15

Retention rate is a useful tool in prescription audits to explain the extent of tolerability and efficacy. For CLB, this rate varies from 61 to 85% in different studies. 3 16 This drug is efficacious in many clinical scenarios. It's effectiveness as add-on therapy in adults and children with refractory focal epilepsy is well established. 17 18 19 20 21 22 Its use in catamenial epilepsy as add on therapy for 10 days during the menstrual cycle also plays an important role. 23

Randomized, double-blind studies in patients with Lennox–Gastaut syndrome showed the efficacy of CLB in reducing the frequency of drop attacks (up to 68.3%) and other seizure types. 24 25

Its use for monotherapy is advocated, despite insufficient evidence. 26 A Canadian study with 235 patients showed that CLB had an equivalent efficacy compared with carbamazepine or phenytoin for children. 27 An open trial in adults also showed favorable results. 28

The drug's antianxiety properties can be very useful in many patients, due to the higher prevalence of this comorbidity. 29 30

Evidence argues against the development of tolerance with CLB treatment. Most patients who initially respond to treatment keep responding at same dosages over the time. Dosage increases during treatment usually reflect efforts to achieve seizure freedom in clinical practice. 31

Long term follow-up of patients with Lennox–Gastaut syndrome (up to 6 years) showed that CLB response was maintained (86%) after 3 years, without significant increases in dosages over the years. 24

The most common side effect is sedation. Irritability can occur. Adverse effects are dose-dependent, however CLB causes less sedation than other 1.4-BZDs.

Polytherapy with carbamazepine and CLB resulted in weight gain in 50% of patients over a period of 6 to 8 months of evaluation. One possible explanation is the increase in carbamazepine epoxide levels due to the concomitant use of CLB. Discontinuation is low, ranging from 3.6 to 16.6%. 24 32

Hepatic failure is extremely rare. There were case reports of toxic epidermal necrosis and Stevens–Johnson syndrome with CLB, but they are rare and most of were taking one or more associated drugs. 5

Abrupt discontinuation of CLB can be associated with withdrawal adverse effects, such as seizures, irritability, restlessness, difficulty in concentration, and insomnia. This can be avoided by gradual discontinuation. But this does not seem to be frequent, as observed for other BZDs.

Data about teratogenicity is insufficient to draw conclusions, though animal data suggests it is a risk factor. 33

For adults, the dosage recommendation is between 5 and 60 mg. An initial dosage of 5 mg once a day is recommended, with increases of 5 to 10 mg each 2 weeks. Target dosage is 10 to 20 mg, depending on patient weight, clinical efficacy, and tolerability, higher doses can be sought (usually above 20 mg/day). In these cases, it can be given in two doses, despite pharmacokinetics suggesting once daily is adequate. 2 16

Also, a higher-evening dose of CLB can improve control in patients with predominantly seizures during sleeping hours or early morning, without increasing side effects. 34

## SHORTAGES AND NEED FOR SWITCHING

Medication shortages pose significant challenges for neurologists and healthcare systems. For epilepsy treatment, one missed dose could lead to breakthrough seizures and associated risks, including sudden unexpected death in epilepsy (SUDEP). 35

They can also make treatment more difficult, create need of extra clinical appointments for orientation and medication switch, increase medication errors, decrease adherence, and cause insecurity to patients and families. 36

Shortages for CLB are not uncommon, and were reported by many countries, including Canada, Turkey, Colombia, United Kingdom, and others. 37 Similarly, there has been a shortage of antiseizure medications in Brazil, with the drug studied here having suffered from this at least three times in recent years.

As previously discussed, CLB has some differences to the other BZDs, and switching it is not an easy or direct task. One critical aspect is determining the equivalent dose to other BZDs.

We reviewed the literature about this topic, with a PubMed search from 1982 a 2025 with the terms “clobazam AND epilepsy AND dosage”. There were 181 results and all titles and abstracts screened, resulting in 28 papers, then all abstracts and texts were read. The papers and references obtained were used for this review. There is a wide variation in dose equivalence described in the literature, presented in Table 1 .

**Table 1:** Dosage equivalence for clobazam switch in different references

Reference	Equivalence	Comments
Sankar et al. 2014 7	10–20 mg CLB = 1 mg CZP	Start clobazam and delay the weaning of clonazepam until significant dose of clobazam is onboard – reduces breakthrough seizures, but increased sedation would be expected.
Asadi-Pooya et al. 2022 38	10–20 mg CLB = 1 mg CZP	Instantly switch can be considered adequate.
Firat et al. 2024 39	10 mg CLB = 1 mg CZP	Seizure frequency was higher during the CZP period, but no status epilepticus. CZP had more side effects.
SPS 40	10 mg CLB = 0.25 mg CZP = 5 mg NTZ = 5 mg DZP	
Canadian League Against Epilepsy 41	10 mg CLB = 1 mg CZP	Start CZP and then further increase dose to total equivalence, with gradual tapering of clobazam if supply allows. For pediatrics, direct substitutions can be made and tapering clobazam is not mandatory.

Abbreviation: SPS, Specialist Pharmacy Service. Note: Adapted from Sankar et al., 7 Asadi-Pooya et al., 38 Firat et al., 39 NHS – Specialist Pharmacy Service, 40 and Canadian League Against Epilepsy. 41

## SURVEY OF BRAZILIAN SPECIALISTS

Due to the actual shortage of CLB and absence of clear recommendations for switch (dosage and need for tapering), we decided to perform a quick survey with Brazilian neurologists, neuropediatricians, neurosurgeons, and neurophysiologists.

The survey was sent online in social media groups by a Google Form (Google LLC.) link from 16 to 29 ^th^ January 2025.

Consent to use the anonymous data for publication was requested before participation.

There were 152 responders, mean age 45.4 years (29–88 years). 75.7% were neurologists (half of them also neurophysiologists) and 19.7% neuropediatricians. Only 14.5% had less than 5 years of experience as specialists (60.5% had > 10 years).

Specialists were asked about the proportion of patients under CLB treatment: 23.7% have less than 10%, 44.7% between 10 and 25%, 25% between 25 and 50% and 6.6% have more than 50%.

**Figure 3:**
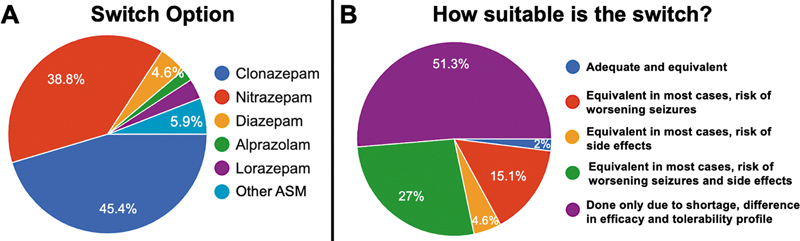
( **A** ) Switch options and ( **B** ) how appropriate is clobazam switching according to experts.

The majority are aware of the CLB shortage (91.4%), and the most frequent alternatives were clonazepam (45.4%), nitrazepam (38.8%), and diazepam (4.6%), as shown in Figure 3A .

The appropriateness of the switch was not deemed adequate and equivalent to 98% of specialists ( Figure 3B ). According to more than half of them, it was done only due to the shortage. Also, concerns about worsening seizures were reported by 93.4%.

**Figure 4:**
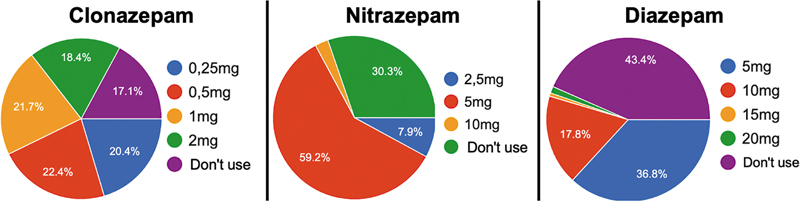
Dose equivalence for clobazam 10 mg switching to clonazepam, nitrazepam, and diazepam.

The dose equivalence for the available BZDs was requested and presented for the most frequent choices in Figure 4 . There is a wide variation in the suggested dose for changing to clonazepam, which is surprising given the lack of consensus on the most suggested alternative.

## DOSAGE AND TITRATION SCHEDULE RECOMMENDATIONS FOR CLOBAZAM SWITCHING

We present here a suggestion based on the literature review, survey results, and authors opinions for the two most frequent and recommended choices, clonazepam and nitrazepam.

The dose equivalence for 10 mg of CLB can vary from 0.5 to 2 mg of clonazepam, usually 1 mg. Each 10 mg of CLB should be reduced by half (5 mg) and start 0.5 mg of clonazepam concomitantly. After 1-week, increase clonazepam to 1 mg and monitor for side effects, specially sedation and seizure recurrence or worsening. If patients present signs of sedation, stop CLB first. If not, stop after another week. Increase the dosage of clonazepam gradually in case of seizure recurrence or worsening. Check for tolerability.

**Figure 5:**
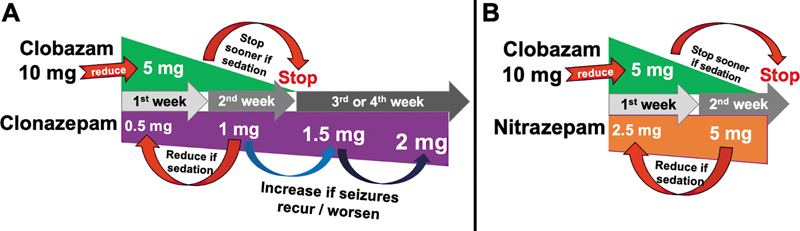
Recommend scheme for switching clobazam to ( **A** ) clonazepam and ( **B** ) nitrazepam.

The equivalence for 10 mg of CLB is usually 5 mg of nitrazepam. Each 10 mg of CLB should be reduced by half (5 mg) and start 2.5 mg of nitrazepam concomitantly. After 1-week, increase nitrazepam to 5 mg and carefully watch for side effects, specially sedation and seizure recurrence or worsening. If patients present signs of sedation, stop CLB first. If not, wait another week before stopping. In case of seizure recurrence or worsening, increasing dosage of nitrazepam is advised, if well tolerated. These recommendations are presented in Figure 5 .

In conclusion, CLB is an effective, well tolerated and safe antiseizure medication, which can be a treatment option for refractory patients as add on therapy. This drug has fewer side effects than other BZDs, especially with a lower rate of sedation. Also, there is a lower risk of developing tolerance. The anxiolytic properties are an extra benefit.

Because of these characteristics, CLB is an essential tool in the epilepsy armamentarium, and shortages are a great risk to patients.

Governments and society must create mechanisms to prevent shortages of critical and unique medications, helping but also regulating the production and stock of pharmaceutical companies.

The available evidence indicates that the alternatives are not equivalent, corroborated by the opinion of experts in our survey. Changing to another BZDs is not easy or safe. Dose equivalence is not well established. Seizure aggravation or recurrence can happen, and side effects can emerge.

The best options for switching according to the literature and expert opinion are clonazepam 1 mg (0.5–2 mg) and nitrazepam 5 for every 10 mg of CLB. We recommend gradual tapering of CLB, with concomitant increase of clonazepam or nitrazepam. Close supervision is advised, checking tolerability and further increases if necessary.
